# Protective Effect of Willow (*Salix babylonica* L.) on Fish Resistance to *Vibrio parahaemolyticus* and *Vibrio alginolyticus*

**DOI:** 10.3390/antibiotics12060989

**Published:** 2023-05-31

**Authors:** Thi Minh Ngoc Mai, Thi Bich Huyen Vu, Minh Ha Le, Thi Thu Hien Nguyen, Thi Thu Hang Trinh, Minh Hai Le, Nguyen Ngoc Tran, Quang Linh Nguyen, Thi Hai Yen Pham, Hoang Nam Pham, Thi Tam Pham

**Affiliations:** 1 Department of Testing and Quality Assurance, Hanoi Open University, 101 Nguyen Hien, Hai Ba Trun, Hanoi 11615, Vietnam; 2 Institute of Biological and Food Technology, Hanoi Open University, B101 Nguyen Hien, Hai Ba Trung, Hanoi 11615, Vietnam; 3 Faculty of Biology, Hanoi National University of Education, 136 Xuan Thuy, Cau Giay, Hanoi 11311, Vietnam; 4 Institute of Natural Products Chemistry, Vietnam Academy of Science and Technology, 18 Hoang Quoc Viet, Cau Giay, Hanoi 10072, Vietnam; 5 Faculty of Fisheries, Vinh University, Vinh 43105, Vietnam; 6 Faculty of Fisheries, University of Agriculture and Forestry, Hue University, Hue City 49116, Vietnam; 7 Department of Life Sciences, University of Science and Technology of Hanoi, Vietnam Academy of Science and Technology, 18 Hoang Quoc Viet, Cau Giay, Hanoi 10072, Vietnam; 8 Department for Scientific Research and International Cooperation, Hanoi Open University, B101 Nguyen Hien, Hai Ba Trung, Hanoi 11615, Vietnam

**Keywords:** *Salix babylonica* L., plant extracts, antibacterial activities, *Vibrio* spp., fish

## Abstract

*Vibrio* spp. cause vibriosis in many saltwater and freshwater aquatic species, such as fish, crustaceans, and mollusks. *Vibrio parahaemolyticus* and *Vibrio alginolyticus* are among the few *Vibrio* species commonly found in infections in fish. This study aimed at investigating the chemical composition and evaluating the antibacterial activities of *Salix babylonica* L. The ethyl acetate (LL2) and methanolic (LL3) extracts were used to evaluate the resistance of strains as *V. parahaemolyticus* LBT6 and VTCC 12233, and two strains of *V. alginolyticus*, NG20 and ATCC 17749, and compared their efficacy with cefotaxime in order to find an alternative to antibiotics in the treatment of vibriosis. The obtained results show that the LL2 extract, with its major components identified as chrysoeriol, luteolin, and β-sitosterol, exhibited a bacteriostatic effect against all the tested strains. In parallel, the LL3 extract, with the four major compounds luteolin-7-*O*-β-D-glucopyranoside, salicin, p-hydroxy benzoic acid, and β-sitosterol-3-*O*-β-D-glucopyranoside, showed significant bactericidal activity against these four strains; the minimal inhibitory concentration (MIC) and minimal bactericidal concentration (MBC) varied from 2.0 to 3.0 μg/mL and from 3.5 to 5.0 μg/mL, respectively. Moreover, the LL3 extract could effectively increase the survival rate of the challenged fish at a dose of 5% (*w*/*w*) for the zebrafish (*Danio rerio*) and 3% (*w*/*w*) for the sea bass (*Lates calcarifer)*. The LL3 extract showed a potential application of *S. babylonica* L. in the prevention and treatment of vibriosis in fish.

## 1. Introduction

Over the past decade, in Vietnam, marine aquaculture has grown strongly in both area and output, and the total area of marine aquaculture in 2010 reached 38,800 hectares; by 2022, it reached 256,479 hectares, and the average growth is 23.3% per year [1]. Like many other countries in the world, marine fish farming in Vietnam has been facing challenges due to various diseases; among these is vibriosis, which is caused by *Vibrio* spp. [2]. In many outbreaks in groupers and sea bass, *V. harveyi* was usually detected at the highest rate, followed by *V. parahaemolyticus*, *V. alginolyticus*, and *V. anguillarum* [3]. In China, a large yellow croaker cultured in Xiangshan Bay was also heavily infected with *V. parahaemolyticus*, *V. harveyi*, and *V. alginolyticus* [4]. In Vietnam, Manh, 2012, reported that 48.4% of infected fish samples with signs of ulcerations, hemorrhages, and skin trauma were infected with *Vibrio* spp., and these bacteria were detected at a relatively high rate in the aquaculture water [5]. Huyen et al., 2022, reported that, among the collected water samples, 47.67% of the samples infected *V. parahaemolyticus*, 45.53% of the samples infected *V. alginolyticus*, 4.65% of the samples infected *V. cholerae*, and 2.33% of the samples infected *V. vulnificus* [6].

Of concern is that the antibiotic resistance of *Vibrio* spp. seems to be seriously increasing. The report of Thu et al., 2019, indicated that the resistance rate of *V. parahaemolyticus* to apramycin was 66.7%, *V. alginolyticus* to oxytetracycline was 60%, and *V. vulnificus* to metronidazole was 66.7% [7]. Cam et al., 2020, reported that *V. parahaemolyticus* is strongly resistant to streptomycin (67%) [8]. Huyen et al., 2022, showed that strains of *Vibrio* spp. isolated from water samples have very high resistance to ampicillin (100%), amoxicillin (98.84%), streptomycin (84.88%), and oxytetracycline (69.77%) [6]. This problem not only leads to limited control of the diseases caused by *Vibrio* spp. but also poses the risk of creating resistant bacterial populations in the aquaculture environment. Therefore, there is a need for effective and safe alternative treatment solutions.

Herbal antibiotics have been applied in aquaculture to prevent and treat diseases in general and vibriosis in particular. Etinosa et al., 2016, reported the anti-vibrio activity of the extracts of acetone and aqueous *Ocimum gratissimum* (Linn) was 47.5% and 30%, respectively [9]. The report of Luo et al., 2022, about the anti-*V. vulnificus* efficacy of oregano essential oil (OEO) indicated that 0.09% OEO could effectively kill *V. vulnificus* in oysters at 25 °C [10]. Ut et al., 2021, reported that guava (*Psidium guajava*), leafflower (*Phyllanthus urinaria* L.) and beach daisy (*Wedelia biflora* (L.) DC) extracts had effective resistance to *Vibrio* spp. isolated from shrimp infected with white feces syndrome in some provinces of the Mekong Delta [11]. In willows (*S. babylonica* L.), Rangel-López et al., 2020, reported the effective resistance to several bacterial species such as *Aeromonas hydrophila*, *Listonella anguillarum*, *Edwarsiella tarda*, and *Streptococcus* [12].

In general, herbal antibiotics have demonstrated high effectiveness in the treatment of diseases in aquatic animals, especially in the context that many antibiotics used in aquaculture are already resistant. For this reason, this study was conducted to provide a solution from willow leaf extract for the control of diseases caused by *Vibrio* spp.

## 2. Materials and Methods

### 2.1. Materials

#### 2.1.1. Plant Materials

The fresh plant leaf samples of *S. babylonica* were collected in autumn at Den Lu Lake, Hanoi City, Vietnam, and were washed individually under running tap water three times (location of sampling point). Leaves were air-dried in the laboratory at room temperature for 10 days. The dried samples were ground well into a fine powder and were stored in air-sealed polythene bags in a refrigerator at 4 °C at the laboratory of the Faculty of Biotechnology, Hanoi Open University.

#### 2.1.2. Bacterial Strains

Four strains of bacteria that cause diseases in aquatic animals were used in antimicrobial assays; they were *V. parahaemolyticus* LBT6 (provided by Research Institute Aquaculture N02), *V. alginolyticus* NG20 (provided by Laboratory of Molecular Biology, Faculty of Biology, Hanoi National University of Education), *V. parahaemolyticus* VTCC 12233 (provided by Center for Biotechnology, Vietnam National University, Hanoi), and commercial strain *V. alginolyticus* ATCC 17749 (Himedia, India).

#### 2.1.3. Experimental Fish

The two experimental fish species in this study were seabass (*Lates calcarifer*), a marine fish that is widely farmed throughout the world (FAO, 2017), and zebrafish (*Danio rerio*), which have been used to study pathogenic *Vibrio* species [13]. Zebrafish with a length of 3–3.5 cm weighing 200–300 mg were purchased at Son Yen Aquatic company, Hoang Hoa Tham Street, Ba Dinh, Hanoi, Vietnam; sea bass measuring 12 cm with an average weight of 1500 mg were provided by Nghe An Aquaculture Breeding Center, Nghe An Province, Vietnam. Before the experiment, fish were confirmed negative for *V. parahaemolyticus* and *V. alginolyticus* by PCR, and then they were domesticated for 7 days.

### 2.2. Methods

#### 2.2.1. Preparation of Plant Extracts

The dried powdered leaves of *S. babylonica* (4.0 kg) were extracted in turn with ethyl acetate and methanol at 50 °C (3 times for each solvent) using heated ultrasonic equipment to produce the ethyl acetate extract (LL2; 93.1 g) and the methanol extract (LL3; 112.7 g). The ethyl acetate and methanol extracts were evaporated under a vacuum and obtained dry extract. The process of soaking, filtration and evaporation is repeated 3 times to completely remove residual solvent. The dried extract is reconstituted in an aqueous solution, then used to study its chemical composition and antibacterial activities.

#### 2.2.2. Chemical Composition Analysis

##### General Procedures

^1^H- and ^13^C-NMR (125 MHz) spectra were recorded on a Bruker AM500 spectrometer at 500 MHz and 125 MHz, respectively. Column chromatography (CC) was carried out on silica gel (0.040–0.063 mm, Merck, Darmstadt, Germany) and YMC RP-C18 resin (150 µm, YMC). Thin-layer chromatography (TLC) was performed on precoated silica gel 60 F254 (Merck). All solvents were distilled before use. Compounds that underwent TLC were observed under UV light at wavelengths 254 nm and 365 nm, and the plate was immersed rapidly in 10% H_2_SO_4_ solution followed by heating.

##### Isolation and Determination

The ethyl acetate extract (LL2; 35 g) was subjected to silica gel CC using a gradient of *n*-hexane-ethyl acetate (30:1, 15:1, 5:1, 2:1, and 0:1; *vol:vol*) to give 5 fractions (E1 to E5). Fraction E2 was further subjected to silica gel column chromatography using *n*-hexane-ethyl acetate (15:1) to yield four subfractions (E2.1 to E2.4). Recrystallization of subfraction E2.2 yielded compound **6** (17.0 mg). Fraction E3 was subjected to a silica gel column using *n*-hexane-acetone (12:1) to give five smaller fractions (E3.1 to E3.5). E3.2 (1.1 g) was further chromatographed through silica gel column chromatography using *n*-hexane-ethyl acetate (8:1) to give 3 subfractions (E3.2.1 to E3.2.3). Fraction E3.2.2 was further chromatographed on a silica gel column with the eluent *n*-hexane-ethyl acetate (4:1) to give three smaller fractions (E3.2.2.1 to E3.2.2.3). Compound **1** (36.5 mg) was obtained from fraction E3.2.2.2 and was chromatographed using an RP-18 column with the eluent methanol and water (1:1). Fraction E4 was further separated on a silica gel column by eluting it with a mixture of *n*-hexane-acetone (10:1) to produce 4 smaller subfractions (E4.1 to E4.3). Compound **2** (42.5 mg) was produced from subfraction E4.2 by using a silica gel column and eluent *n*-hexane-ethyl acetate (2:1).

The methanol extract (LL3; 40 g) was subjected to silica gel CC using a gradient of ethyl acetate-methanol (100:0 to 0:100; vol: vol) to give 6 fractions (M1 to M6). M1 was separated on a silica gel column using dichloromethane-methanol (15:1) to obtain 4 smaller fractions (M1.1 to M1.4). Subfraction M1.1 was further separated on a silica gel column using dichloromethane–methanol (10:1) to yield compound **7** (13.5 mg). Fraction M1.2 was then separated on a silica gel column with the eluent *n*-hexane and ethyl acetate (2:1) to give compound **5** (9.5 mg). Fraction M2 was further separated on a silica gel column using dichloromethane-methanol (8:1) to obtain four subfractions (M2.1 to M2.4). Fraction M2.2 was then separated on a silica gel column using dichloromethane, methanol, and water (7:1:0.1) to give three smaller fractions (M2.2.1 to M2.2.3). Compound **4** (1287 mg) was obtained after purifying fraction M2.2.2 using RP-18 column and methanol-water (2:1). The first was subject to a silica gel column with dichloromethane–methanol-water (9:1:0.1) as eluent to produce five subfractions (M3.1 to M3.5). Compound **3** (12.8 mg) was produced from fraction M3.2.3 using an RP-18 column with methanol-water (2:1) as the eluent.

#### 2.2.3. Minimal Inhibition Concentration (MIC) and Minimal Bactericidal Concentration (MBC) Determination

The index of the minimum inhibitory concentration (MIC) and the minimum bactericidal concentration (MBC) of the extracts against the studied bacteria was determined using the method of NaPhatthalung et al., 2017. The extracts (LL2 and LL3) were diluted in dimethylsulfoxide (DMSO, Sigma Aldrich, St. Louis, MO, USA) solution and Luria Broth media (LB, Himedia, India) 1% NaCl medium to obtain concentrations of 0–15 mg/mL and were added to the wells of a 96-well plate. The concentration of DMSO in each well did not exceed 5% to ensure that bacterial growth was not inhibited.

Bacteria were cultured, achieving a concentration of 2 × 10^6^ CFU/mL, and were placed in a 96-well plate containing willow extracts. Each well consisted of 100 μL of bacterial broth and 100 μL of extracts at different dilutions. The control wells were without extracts.

The 96-well plate was incubated at 28 °C for 24 h. The number of bacteria was determined by culturing them on thiosulfate citrate bile salts sucrose agar (TCBS, Himedia, India). The MIC value was the lowest concentration in the experimental concentration range of the extracts that could inhibit bacterial growth. The MBC value was the lowest concentration in the concentration range of the extracts that could kill all the bacteria in the well (no colonies appeared on the TCBS agar plate). The experiments were repeated 3 times.

#### 2.2.4. Time-Kill Study

Time-kill study was conducted to determine the kinetics of the bactericidal effect of LL3 extracts on *V. parahaemolyticus* and *V. alginolyticus*. This method was carried out according to the method of Na-Phatthalung et al., 2017 [14]. The bacteria was prepared to a concentration of 2.5 × 10^6^ CFU/mL. Each tube was supplemented with 1350 µL of LB containing LL2 or LL3 extract at concentrations of 0 × MIC, 0.25 × MIC, 0.5 × MIC, MIC, or 2 × MIC and 150 µL of bacteria, respectively. Tubes were shaken at 160 rpm at 28 °C. A total of 100 µL of the experimental solution in each tube was spread on TCBS medium in turn at different times (0 h, 4 h, 8 h, 12 h, and 24 h) to detect the number of bacteria. The experiment was repeated 3 times for each sample.

#### 2.2.5. Trial of Willow Extract on Fish Challenged with *Vibrio* sp.

This method was implemented according to Mohammed et al., 2016 [15].

Fish were randomly arranged into 10 HDPE plastic tanks with capacities of 300 L, including 5 tanks for sea bass, each tank containing 50 fish, and 5 tanks of zebrafish with 100 fish in each tank. Experimental fish were reared in the same culture conditions, including aeration mode, temperature conditions (25–30 °C), and type of food (Cargill). During the experiment, the water was changed daily; the amount of water changed was 30%/tank. Fish feed was mixed with extract solution at concentrations of 0.1%, 1%, 3%, and 5%. The control batch was not supplemented with extracts. Zebrafish and sea bass were divided into study groups and were fed with mixed feed at a defined concentration of 2% of their body weight for 2 weeks. After 2 weeks of treatment, experimental fish were challenged with *V. parahaemolyticus* and *V. alginolyticus* at a concentration of 5 × 10^6^ CFU/mL; zebrafish were injected with a volume of 50 µL/fish, and sea bass were injected with a volume of 200 µL/fish. The survival rates of fish were determined over different time periods (7 days for zebrafish; 10 days for sea bass). During the postinfection follow-up period, fish were still fed the experimental diet. The experiment was repeated 3 times.

## 3. Results

### 3.1. Isolation of Major Chemical Components of Extracts

**Figure 1:**
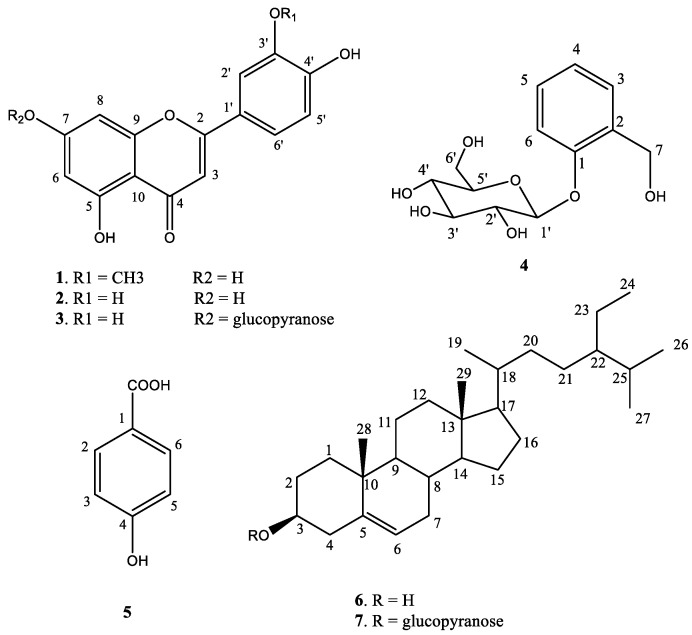
Structures of isolated compounds.

A chemical investigation into LL2 led to the isolation of three compounds: chrysoeriol (1), luteolin (2), and *β*-sitosterol (6). Chrysoeriol and luteolin were identified as the main compounds of LL2 with high isolation efficiencies of 0.1% and 0.12% (compared to LL2’s weight), respectively. Meanwhile, a chemical study on LL3 led to the isolation of four compounds: luteolin-7-*O*-*β*-D-glucopyranoside (3), salicin (4), p-hydroxy benzoic acid (5), and *β*-sitosterol-3-*O*-*β*-D-glucopyranoside (7). Salicin was found to be the major compound of LL3, with the highest isolation efficiency of 0.32% (compared to LL3’s weight). Their structures (Figure 1) were identified by analyzing their 1D and 2D-NMR spectra in comparison with the data previously reported [16,17,18,19,20,21].

### 3.2. Antibacterial Assay

The MIC and MBC values of LL2 and LL3 were investigated to determine their ability to resist the *V. parahaemolyticus* and *V. alginolyticus* strains. The results in Table 1 show that the MIC values of LL2 were 2.0–5.0 µg/mL and that the MBC was 9.5–17.5 µg/mL; LL3 had MIC and MBC values of 2.0–3.0 µg/mL and 3.5–5.0 µg/mL, respectively. In this study, cefotaxime, an antibiotic that is strongly sensitive to *Vibrio* spp. and commonly used in the treatment of vibriosis in aquatic animals, was used as a control; the obtained results show that the MIC and MBC values of cefotaxime were significantly lower than those of the willow extracts, and the values obtained were 0.1–0.36 µg/mL and 0.36–3.0 µg/mL, respectively.

According to Canillac and Mourey, 2001, if the ratio of MBC/MIC is ≤4, the extract has a bactericidal effect, and if the ratio of MBC/MIC is >4, the extract has a bacteriostatic effect [22]. The results in Table 1 show that LL2 is bacteriostatic and that LL3 is bactericidal. Therefore, the LL3 extract was selected to consider its resistance to several strains of *Vibrio* sp. and the effectiveness of treatment for fish challenged with the above bacterial strains.

**Figure 2:**
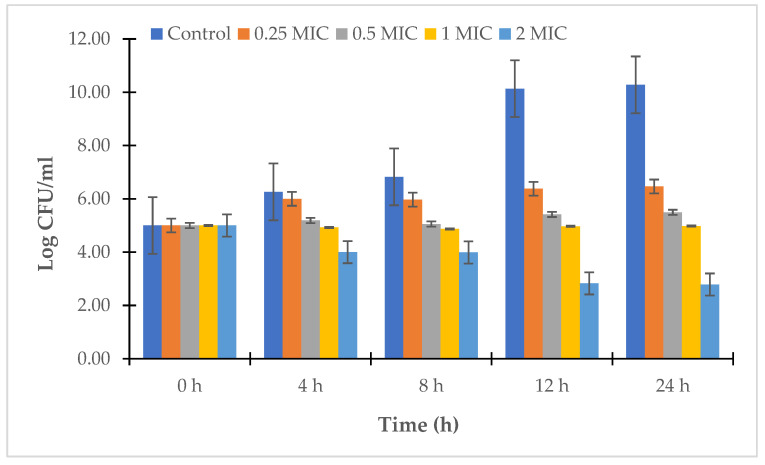
Time-kill kinetics of LL3 against *V. parahaemolyticus* VTCC 12233.

**Figure 3:**
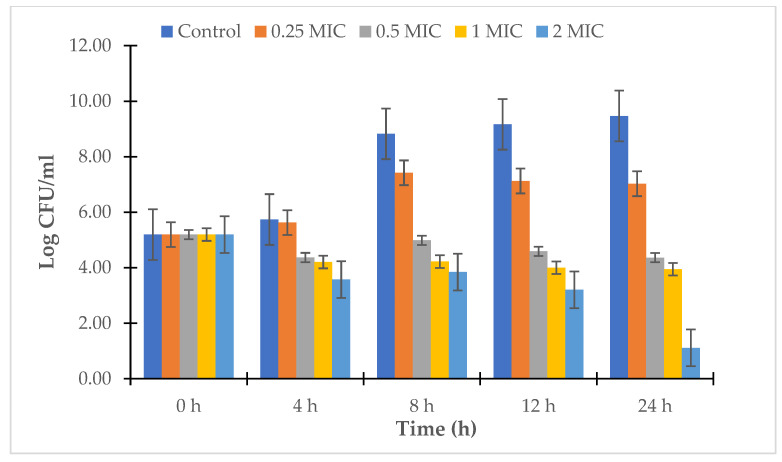
Time-kill kinetics of LL3 against *V. parahaemolyticus* LBT6.

The results in Figure 2 and Figure 3 show that LL3 was effective in significantly reducing the concentration of *V. parahaemolyticus* at a dose of 2 × MIC; the concentration of *V. parahaemolyticus* VTCC 12233 and *V. parahaemolyticus* LBT6 strongly decreased after 24 h of treatment with the LL3 extract, the bacterial concentration was only between 10^2.78^ and 10^1.11^ CFU/mL. Meanwhile, the doses of 0.5 × MIC and 1 × MIC caused a similar decrease in the bacterial concentration after 24 h of treatment, and the bacterial concentrations were 10^4.36^–10^3.94^ CFU/mL and 10^5.49^–10^4.98^ CFU/mL, respectively, for *V. parahaemolyticus* LBT6 and *V. parahaemolyticus* VTCC12233.

**Figure 4:**
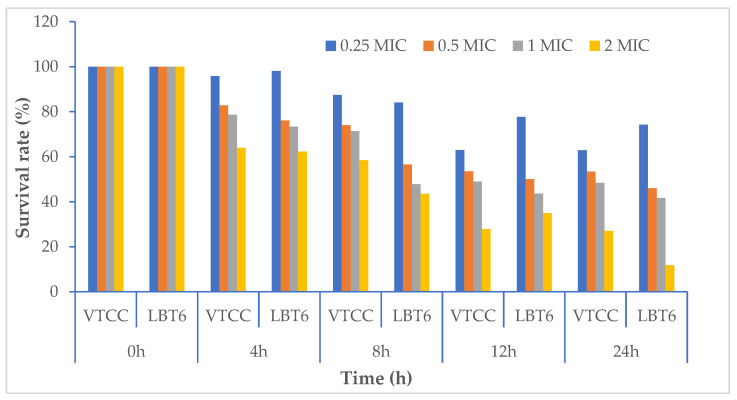
The survival rate of *V. parahaemolyticus* VTCC 12233 and *V. parahaemolyticus* LBT6 following treatment with LL3.

Comparing the survival rates of the bacteria treatment with LL3 and the control using DMSO showed that, after 24 h, the survival rates of *V. parahaemolyticus* LBT6 and *V. parahaemolyticus* VTCC 12233 when treated with the dose of 2 × MIC was 11.77% and 27.08%, respectively; the survival rates of *V. parahaemolyticus* LBT6 and *V. parahaemolyticus* VTCC 12233 were 41.67% and 46.07%, 74.21% and 48.43%, 53.42% and 62.87% corresponding to the treatment doses of 1 × MIC, 0.5 × MIC, and 0.25 × MIC (Figure 4).

**Figure 5:**
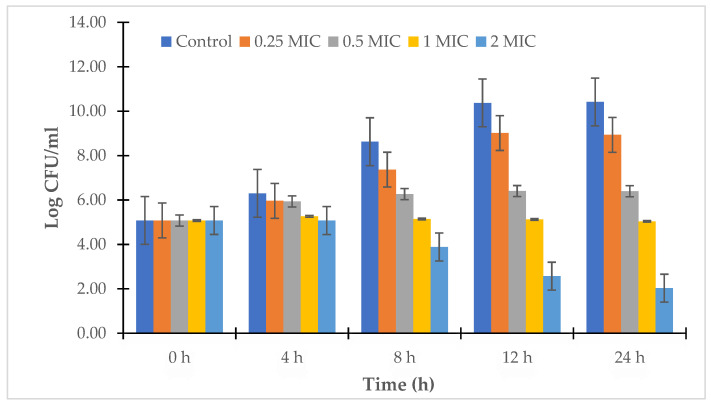
Time-kill kinetics of LL3 against *V. alginolyticus* ATCC 17749.

**Figure 6:**
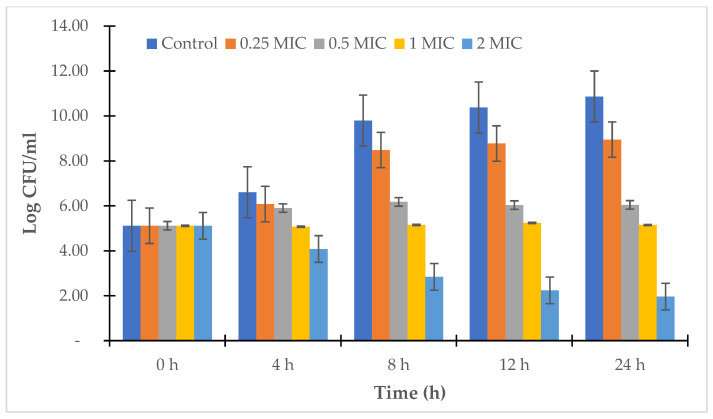
Time-kill kinetics of LL3 against *V. alginolyticus* NG20.

**Figure 7:**
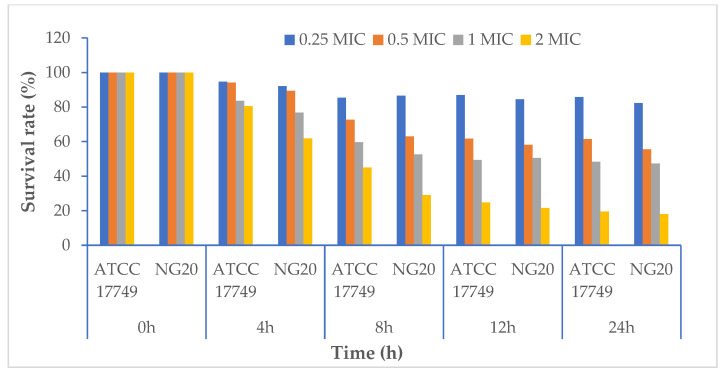
Survival rate of *V. alginolyticus* ATCC 17749 và *V. alginolyticus* NG20 following treatment with LL3.

For the strains *V. alginolyticus* ATCC 17749 and *V. alginolyticus* NG20, the LL3 extract also reduced the bacterial density of the culture, as well as the survival rates of the strains. Specifically, after 24 h of culture, the bacterial densities of *V. alginolyticus* ATCC 17749 were 10^8.93^, 10^6.4^, 10^5.04^, and 10^2.03^ CFU/mL, and the densities of the *V. alginolyticus* NG20 strain were 10^8.95^, 10^6.04^, 10^5.15^, and 10^1.96^ CFU/mL corresponding to the treatment doses of 0.25 × MIC, 0.5 × MIC, 1.0 × MIC, and 2.0 × MIC (Figure 5 and Figure 6); the survival rates of *V. alginolyticus* ATCC 17749 were 85.77%, 61.43%, 48.41%, and 19.52%, and those of *V. alginolyticus* NG20 were 82.36%, 55.61%, 47.37%, and 18.08%, respectively, for the above treatment doses (Figure 7).

### 3.3. The Effects of Willow Extracts on Fish Challenged with Vibrio sp.

**Figure 8:**
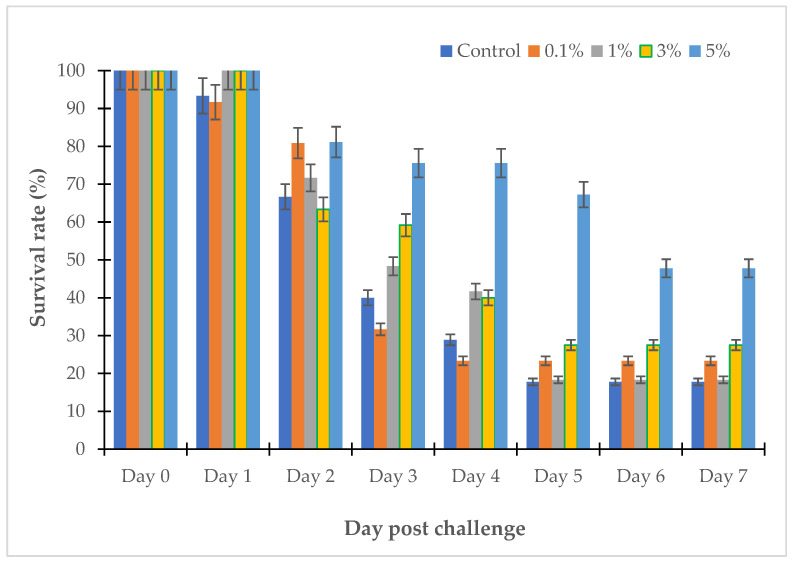
Survival rate of LL3-treated zebrafish challenged with *V. parahaemolyticus* LBT6.

**Figure 9:**
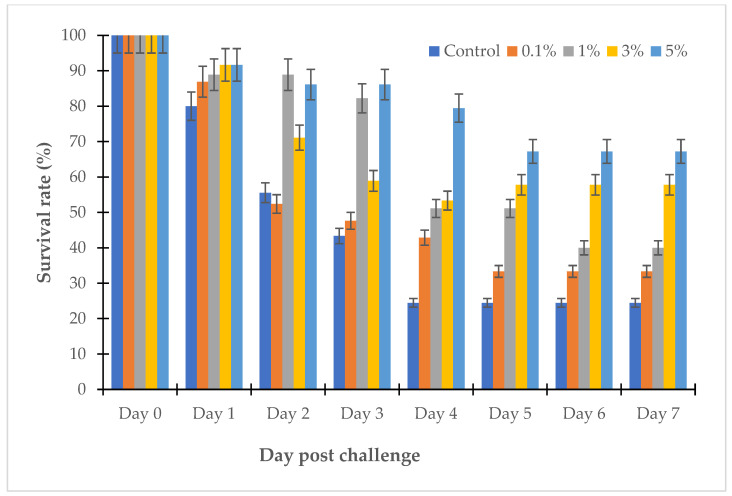
Survival rate of LL3-treated zebrafish challenged with *V. alginolyticus* NG20.

**Figure 10:**
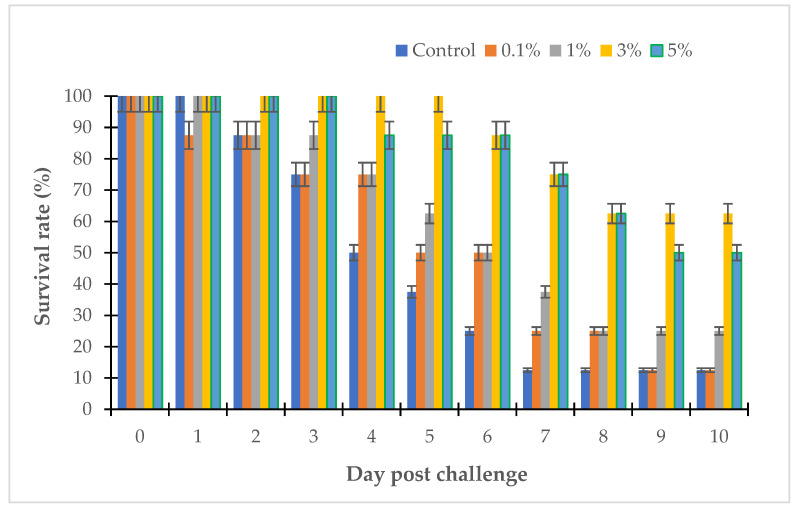
Survival rate of LL3-treated sea bass challenged with *V. parahaemolyticus* LBT6.

**Figure 11:**
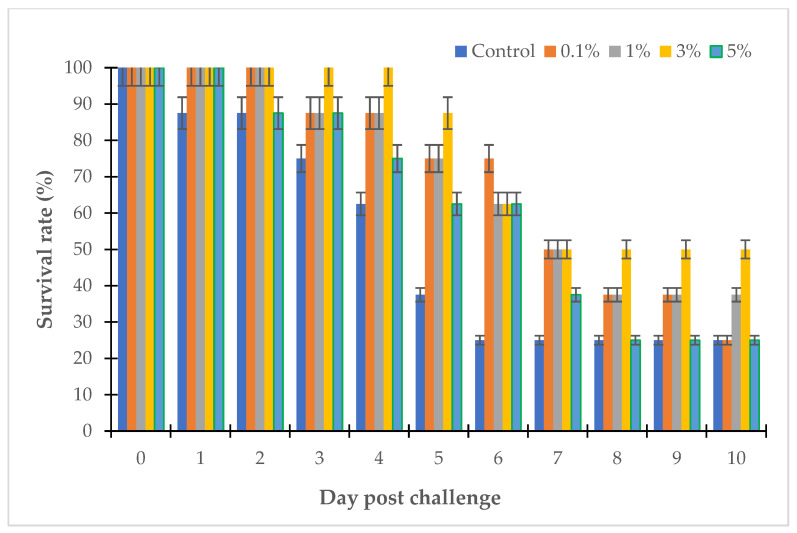
Survival rate of LL3-treated sea bass challenged with *V. alginolyticus* NG20.

The MIC and the MBC are important values that reflect the activity of antibacterial substances; however, they do not show the complete activity of antibiotics in clinical practice. The clinical effectiveness of antimicrobials depends on the pharmacokinetic properties of the antibiotic. In this study, the therapeutic effect of the LL3 extract was evaluated in zebrafish and sea bass challenged with several strains of *Vibrio* sp., and the results are shown in Figure 8, Figure 9, Figure 10 and Figure 11.

The survival rates of the zebrafish challenged with *V. parahaemolyticus* LBT6 and *V. alginolyticus* NG20 in the tanks treated with LL3, as well as the controls, gradually decreased during the experiment. In the control tanks, after 7 days, the survival rates of the zebrafish challenged with *V. parahaemolyticus* LBT6 and *V. alginolyticus* NG20 were 17.78% and 24.44%, respectively; the challenged fish showed sores and necrosis at the injection site; in the treatment tanks. After 7 days, the survival rates of the fish challenged with *V. parahaemolyticus* LBT6 and *V. alginolyticus* NG20 were 23.33%, 18.33%, 27.5%, and 47.78% and 33.33%, 40%, 57.78%, and 67.22% corresponding to the doses of 0.1%, 1%, 3%, and 5% of LL3. The survival rate of the challenged zebrafish treated with 5% of LL3 was the highest (Figure 8 and Figure 9).

After 10 days of the experiment, the survival rates of the sea bass challenged with *V. parahaemolyticus* LBT6 and *V. alginolyticus* NG20 were 12.5% and 25%, respectively (Figure 10 and Figure 11). Performing the treatment with the LL3 extract on the sea bass challenged with *V. parahaemolyticus* LBT6 and *V. alginolyticus* NG20 gave the following results: corresponding to the doses of 0.1%, 1%, 3%, 5% of LL3, the survival rates of the treatment fish were 12.5%, 25%, 62.5%, 50% and 25%, 37.5%, 50%, 25% (Figure 10 and Figure 11). The survival rate of the challenged sea bass reached the highest value in the trial with a treatment dose of 3% of LL3.

## 4. Discussion

*S. Babylonica* is a tree widely distributed in Africa, North America, Europe and Asia. This plant is used in traditional medicine to treat various musculoskeletal pain, inflammation and fever. Many studies have shown the potential of this plant in antifungal, bacteriostatic [23], HIV-inhibitory [24], antioxidant [25,26], and anti-cancer [27] applications. Salicin is the main substance metabolism of *S. Babylonica*, and it has pharmacological activity. In the gastrointestinal tract, this compound is hydrolyzed to form salicyl and D-glucose. After absorption in the host, it is oxidized to salicylic acid, which has cyclooxygenase inhibitory activity (COX I, II) [28]. *The p*-hydroxybenzoic acid of *S. Babylonica* was proven to be effective against some types of Gram-positive and Gram-negative bacteria [29]. The other components of this plant as chrysoeriol, luteolin, luteolin-7-*O*-*β*-D-glucopyranoside, *β*-sitosterol, and *β*-sitosterol-3-*O*-*β*-D-glucopyranoside was also reported antibacterial activity [30,31,32,33]. Our study also identified compounds similar to previous reports, which may be responsible for the antibacterial activities of the plant extracts.

Research by Wahab et al., 2018, indicates that *S. Babylonica* extract is effective against resistant gram-positive bacteria (*S. aureus*), gram-negative bacteria (*E. coli*, *K. pneumoniae*, and *P. aeruginosa*), and pathogenic fungi such as *C. albicans* [34]. González-Alamilla et al., 2020 also determined the antibacterial ability of willow extract, with a concentration of 50 mg/mL, extract able to inhibit *E. coli*, *Salmonella typhi*, *Salmonella cholerasuis*, *P. aeruginosa*, L. *monocytogenes*, and *S. aureus*; *B. subtilis* was inhibited at a concentration of 25 mg/mL [35].

For pathogenic bacteria in aquatic animals, Rangel-López et al., 2020, demonstrated that the *S. babylonica* hydroalcoholic (SbHE) extract was resistant to *A. hydrophila* CAIM347, *Listonella anguillarum* CAIM 763, *Edwardsiella tarda* CAIM 1875, and *S. iniae* CAIM527 [12]. In this study, the results determining its activity against some strains of *V. parahaemolyticus* and *V. alginolyticus*, as well as its effectiveness in treating sepsis in fish caused by these strains, showed that the willow leaf extract has potential in the treatment of diseases. The bioactive compounds of willow leaves analyzed above are known for antibacterial properties; therefore, it is possible to develop preparations to treat bacterial infections in aquatic animals.

## 5. Conclusions

The results obtained from this study indicate that the LL3 extract of *S. babylonica* was significantly reducing the density of the *V. parahaemolyticus* and *V. alginolyticus* strains and effectively increased the survival rates of the challenged zebrafish and sea bass. From these results, we can initially conclude that willow extracts have potential applications in the prevention and treatment of vibriosis in fish.

## Figures and Tables

**Table 1:** Minimal inhibition concentration (MIC) and minimal bactericidal concentration (MBC) of LL2 and LL3 against *V. parahaemolyticus* and *V. alginolyticus*.

Bacteria	LL2 (μg/mL)		LL3 (μg/mL)		Cefotaxim (μg/mL)	
	MIC	MBC	MIC	MBC	MIC	MBC
*V. alginolyticus* ATCC 17749	3.75	17.5	3.0	5.0	1.0	3.0
*V. alginolyticus* NG20	3.5	17.5	2.0	3.5	2.0	1.0
*V. parahaemolyticus* VTCC 12233	2.0	9.5	2.0	3.75	2.0	1.0
*V. parahaemolyticus* LBT6	3.5	12.0	2.0	3.75	0.56	1.5

## Data Availability

Not applicable.
